# TWO TALES OF TRANSLATION: EVIDENCE-BASED CAREGIVER PROGRAMS THROUGH COMMUNITY-BASED ORGANIZATIONS

**DOI:** 10.1093/geroni/igz038.1653

**Published:** 2019-11-08

**Authors:** David Coon, Carol Whitlatch

**Affiliations:** 1 Arizona State University, Phoenix, Arizona, United States; 2 Benjamin Rose Institute, Cleveland, Ohio, United States

## Abstract

This presentation reports findings from on different translation approaches that embedded into the community two evidence-based interventions for family caregivers of people with dementia. CarePRO was embedded into the community after completion of a clinical trial, whereas EPIC was embedded from the program’s inception. CarePRO is a group and telephone coach call intervention targeting family caregivers and EPIC is a group dyadic intervention for both early-stage people and their care partners. Available in English and Spanish, these programs are still being delivered across two states through community-based organizations. Findings from both CarePRO and EPIC demonstrate significant levels of participant benefit (e.g., reduced negative mood states, negative coping strategies, and negative network interactions as well as increased positive mood states, self-efficacy, care preparedness). Presenters will share lessons learned from the translation process regarding design modifications, training and supervision of interventionists, participant recruitment and retention, and adaptation of outcome assessments.

